# Concomitance of persistent primitive olfactory artery, accessory middle cerebral, and early bifurcated anterior cerebral arteries

**DOI:** 10.1007/s00276-025-03647-3

**Published:** 2025-05-05

**Authors:** George Triantafyllou, Panagiotis Papadopoulos-Manolarakis, Fabrice Duparc, George Tsakotos, Maria Piagkou

**Affiliations:** 1 Department of Anatomy, School of Medicine, Faculty of Health Sciences, National and Kapodistrian University of Athens, 75 Mikras Asias str, Goudi, Athens, 11527 Greece; 2 Department of Neurosurgery, General Hospital of Nikaia-Piraeus, Athens, Greece; 3 Department of Anatomy, Faculty of Medicine-Pharmacy, University of Rouen-Normandy, Rouen, France

**Keywords:** Persistent primitive olfactory artery, Accessory middle cerebral artery, Early bifurcated anterior cerebral artery, Anatomy, Variation

## Abstract

The cerebral arterial circle is susceptible to various developmental anomalies that may persist in adulthood. Notably, the persistent primitive olfactory artery (PPOA) is a rare variation, with a reported prevalence of 0.14%, and it is infrequently paired with other vascular anomalies. This imaging report details a unique instance involving a 65-year-old male patient presenting a rare combination of a left-sided PPOA, a left-sided accessory middle cerebral artery (AMCA), and an early bifurcation of the anterior cerebral artery (ACA). The arterial variations were identified utilizing computed tomography angiography (CTA). The PPOA, characterized by its distinctive hairpin turn and nasal branch, runs parallel to the A2 segments, while the AMCA emerges as an anomalous branch from the ACA. Comprehending such configurations is essential for preoperative planning in anterior skull base surgeries due to the associated risks of aneurysm formation and potential intraoperative complications. This case underscores the significance of recognizing rare vascular anomalies to mitigate surgical risks and enhance patient outcomes.

## Introduction

The adult cerebral arterial circle is established through the anastomosis of the terminal branches of the internal carotid artery (ICA) along with the vertebrobasilar system, which includes the anterior, middle, and posterior cerebral artery (ACA, MCA, and PCA) [7].

However, these vessels are shaped by other embryonic arteries that undergo regression or fusion throughout embryological development to establish the adult mature arterial system. The embryonic ICA gives rise to rostral and caudal divisions that supply the brain. The rostral division is identified as the primitive olfactory artery, which supplies the prosencephalon and terminates in the nasal fossa [5]. In rare instances, this artery may persist in adulthood, resulting in the formation of the persistent primitive olfactory artery (PPOA), which has been reported to have a prevalence of 0.14%, according to a large retrospective study [11].

Contemporary literature infrequently documents the coexistence of rare variants of the cerebral arterial circle. This report delineates a distinctive arterial anatomy characterized by the amalgamation of various variants.

### Anatomic variation

During an angiographic study utilizing a computed tomography angiography (CTA) archived dataset, the anatomical characteristics of a 65-year-old male patient were meticulously examined. This dataset was procured from the General Hospital of Nikaia-Piraeus after obtaining ethical approval from the relevant authorities (protocol number: 56485, date of approval: 13 November 2024). The scans were documented using Horos software version 3.3.6 (Horos Project). In alignment with findings from prior studies [8], evidence was obtained through the multiplanar reconstruction of axial, coronal, and sagittal slices alongside three-dimensional volume reconstruction.

The bilateral ICAs typically branch into the left and right ACAs and MCAs within the anterior circulation.

On the left side, the A1 segment measured 14.2 mm in length and 1.83 mm in diameter. At the junction of the A1 and A2 segments (A1-A2), an accessory branch (diameter of 1.2 mm) was identified, originating from this point and following a course along the left MCA; consequently, this branch was recognized as an accessory left MCA (AMCA) originating from the ACA.

**Fig. 1:**
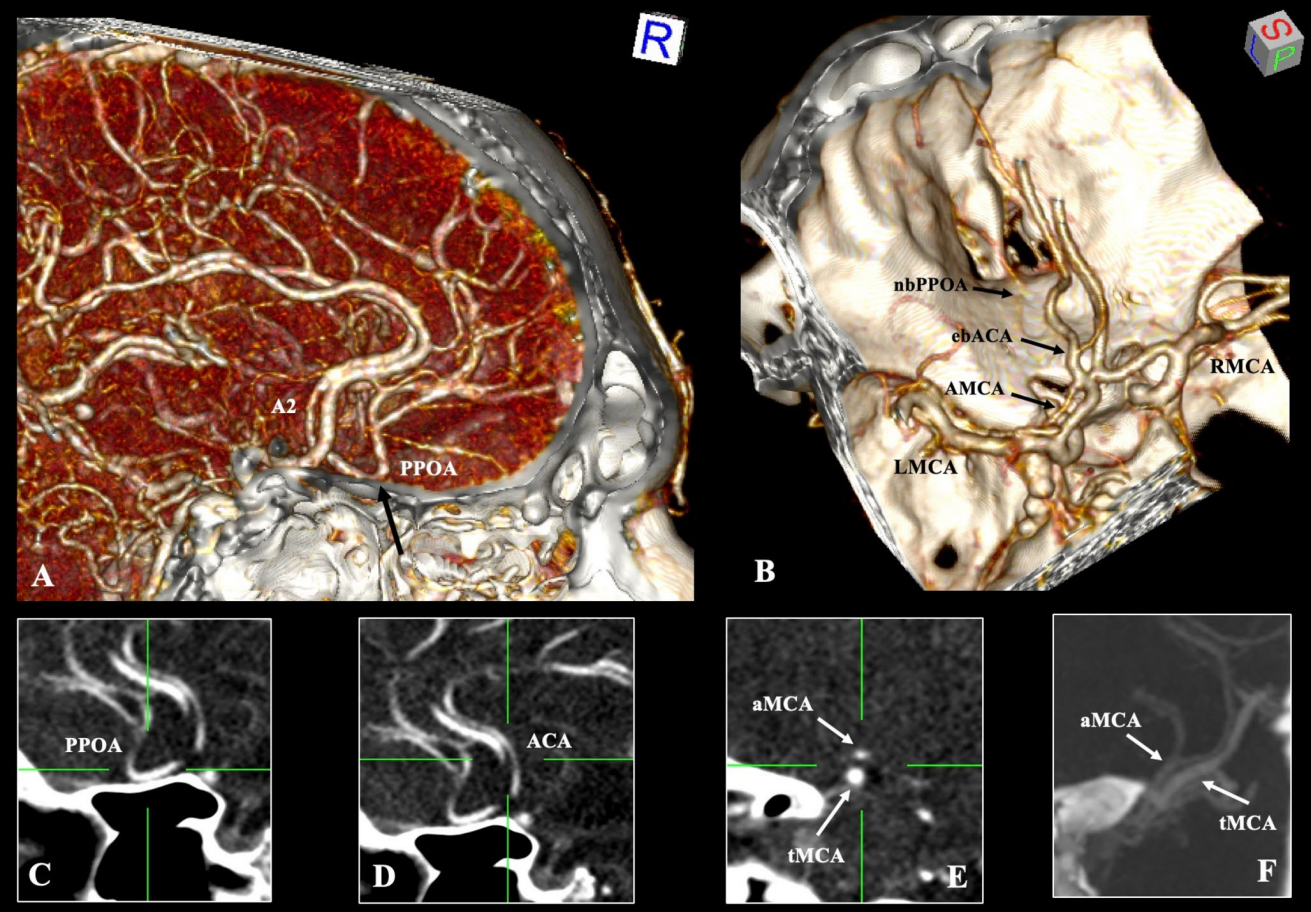
Three-dimensional reconstruction of the cerebral arterial circle of the male patient (**A**, **B**). The persistent primitive olfactory artery (PPOA) is indicated through sagittal slices (**C**). The typical anterior cerebral arteries (ACA) in sagittal slices (**D**). The accessory middle cerebral artery (aMCA) in sagittal and coronal slices (**E**, **F**). tMCA- typical middle cerebral artery, LMCA- left middle cerebral artery, RMCA- right middle cerebral artery, ebACA- early bifurcated anterior cerebral artery, nbPPOA- nasal branch of the persistent primitive olfactory artery

The A2 segment, measuring 2.6 mm long, bifurcated into a primary branch with a diameter of 1.6 mm and followed the conventional trajectory of the contralateral (right-sided) A2 segment. In contrast, the second branch (with a diameter of 1.8 mm) pursued an anteroinferior trajectory towards the cribriform plate of the ethmoid bone, gave rise to a nasal branch, executed a hairpin turn, and continued in parallel orientation with the two A2 segments. Therefore, this branch is classified as a PPOA variant (Fig. 1).

The posterior circulation of the patient was free of variants.

## Discussion

The present anatomical-imaging report shows the rare variant of a unilateral PPOA combined with a left AMCA and early bifurcation of the ACA. During the fifth week of gestation, the ACA develops from the primitive olfactory artery (POA) [3]. Typically, it regresses to form the recurrent artery of Heubner; however, when this artery fails to develop, a PPOA arises and has a hairpin turn due to the posterosuperior location of the typical distal ACA (dACA) [3]. Regarding the development of an AMCA, the typical MCA develops after the ACA. Therefore, an AMCA could be seen as an anomalous early ramification of an early branch of the MCA [3]. The AMCA is regarded as the supernumerary vessel that originates from the ACA and follows a trajectory similar to the typical MCA. Recently, Sharma et al. [6] calculated the pooled prevalence at 1%, indicating that this variation is infrequent. An increased risk of aneurysm has been associated with the AMCA origin due to abnormal hemodynamic stress on the vessel’s wall [6].

Uchino et al. [11] conducted one of the few published studies on the PPOA morphological variability using MRAs. They reported a prevalence of 0.14% among 3,491 patients. Only one patient had bilateral PPOA, indicating that bilateral occurrence is even rarer. Additionally, one patient was associated with an aneurysm located at the sharp bend in the PPOA course [11]. Kim and Lee [2] recorded the PPOA variations in their sample using CTAs. They identified eight patients with PPOA among the 3,067 patients, resulting in a prevalence of 0.26% [2]. Five categories of PPOA have been delineated: Type 1- typical configuration (similar to the present case), Type 2- a small artery that connects with the ethmoidal artery, Type 3- supplies both the dACA and the ethmoidal artery, Type 4- a continuation to the AMCA, and Type 5- connects with the A3 segment without forming a hairpin turn [9]. Clinically, the PPOA is associated with the presence of an aneurysm on the variant artery. It has been successfully treated in the past with a fronto-temporal craniotomy or an interhemispheric approach. Most of the aneurysms were clipped without causing any further neurological deficits [2]. It can be asymptomatic but may be associated with aneurysms or cerebrovascular anomalies due to altered hemodynamics. The hairpin turn of the PPOA may influence hemodynamics and represent a risk factor for aneurysm formation at this location [11]. Although this vessel is rarely encountered, it is crucial to identify it before anterior skull base surgeries. Several surgical procedures are performed at the anterior skull base [4]. Intraoperatively, the surgeon should recognize its presence to prevent injury and bleeding, while it may also supply the arteriovenous fistula located in the region [2].

The presence of the PPOA is rare, while the configuration and other cerebral arterial variants create a unique morphology. The current case describes the coexistence of a PPOA with an AMCA and an early bifurcated ACA. Radoi et al. [5] recorded combined rare PPOA variants with azygos pericallosal artery during CTA of a 71-year-old male patient. Uchino and Mochizuki [9] identified the PPOA and the AACA, which also had an early bifurcation. Uchino and Ogiichi [10] reported the sporadic bilateral PPOA presence with an accessory ACA during MRA of a 48-year-old male patient. Uchino and Ishihara [8] reported the coexistence of PPOA (type 4 variant) with an AMCA and ACA fenestration during the MRA of a 40-year-old female patient. Recently, Endo et al. [1] identified the PPOA with an AMCA and a partial duplication of the anterior communicating artery. Therefore, these case reports highlight that the combination of rare arterial variants is scarcely described in the current literature (Table 1).

**Table 1:** Literature summary of the coexistence of persistent primitive olfactory artery (PPOA) with other cerebral arterial variants. M- male, F- female, ACA- anterior cerebral artery, MCA- middle cerebral artery, AComA- anterior communicating artery

Authors (Year)	Imaging	Sex/Age	PPOA Characteristics	Combined with
Radoi et al. (2021) [5]	CTA	M71	Unilateral (left)	Azygos pericallosal ACA
Uchino and Mochizuki (2021) [9]	MRA	F74	Unilateral (left)	Early bifurcation of accessory ACA
Uchino and Ishihara (2021) [8]	MRA	F40	Unilateral (left)	Accessory MCA and distal ACA fenestration
Uchino and Ogiichi (2022) [10]	MRA	M48	Bilateral	Accessory ACA
Endo et al. (2025) [1]	MRA	F46	Bilateral	Accessory MCA and AComA partial duplication
Current Case Report	CTA	M65	Unilateral (left)	Accessory MCA and ACA early bifurcation

In conclusion, we have delineated the combination of a left-sided PPOA with a left-sided AMCA alongside an early bifurcation of the ACA. These variations contributed to the unique cerebral arterial circle observed in the male patient. Preoperative awareness of such arterial variants via CTA or MRA is imperative prior to undertaking surgical or endovascular interventions, given that these infrequent variations have also been associated with the formation of aneurysms.

## Data Availability

No datasets were generated or analysed during the current study.
